# Presence and Evolution of Radiological Changes at 6 and 12 Months After COVID-19 Pneumonia and Their Risk Factors

**DOI:** 10.3390/medicina61030382

**Published:** 2025-02-22

**Authors:** Celia Roig-Martí, Antonio Navarro-Ballester, María-Pilar Fernández-García, Ignacio Pérez-Catalán, Ana Segura-Fábrega, María Varea-Villanueva, Sofía Folgado-Escudero, Germán Herrero-Rodríguez, Elena Domínguez-Bajo, Sergio Fabra-Juana, María-José Esteve-Gimeno, María-Lidón Mateu-Campos, Jorge Usó-Blasco, José-Manuel Ramos-Rincón

**Affiliations:** 1 Internal Medicine Department, Castellón General University Hospital, 12004 Castellón, Spain; 2 Radiology Department, Castellón General University Hospital, 12004 Castellón, Spain; 3 Intensive Care Unit, Castellón General University Hospital, 12004 Castellón, Spain; 4 Internal Medicine Department, Alicante General University Hospital, 03010 Alicante, Spain; 5 Alicante Institute of Health and Biomedical Research (ISABIAL), 03010 Alicante, Spain; 6 Clinical Medicine Department, Miguel Hernández University of Elche, 03202 Elche, Spain

**Keywords:** COVID-19, tomography, pulmonary fibrosis, SARS-CoV-2, pneumonia

## Abstract

*Background and Objectives*: The pulmonary sequelae of COVID-19 and their evolution are of interest to the scientific community. We aimed to determine the radiological changes at 6 and 12 months after COVID-19 pneumonia, its evolution and its risk factors. *Materials and Methods*: This retrospective longitudinal study included adults admitted for COVID-19 pneumonia from 1 March 2020 to 30 April 2021 who had a high-resolution computed tomography (HRCT) scan at 6 months and 12 months after hospital discharge. The primary outcome was the appearance of radiological abnormalities on HRCT and the number of lung segments affected by them at 6 and 12 months, while the main explanatory variables were about the disease course, analytical parameters and treatment. *Results*: This study included n = 108 patients, with a mean age of 64 years. There was a decrease in the percentage of patients presenting parenchymal (93.5% to 88.9%, *p* < 0.001) and reticular (63% to 62%, *p* < 0.001) patterns on HRCT at 12 months compared to 6, and an increase in those presenting a fibrotic pattern (62% to 63.9%, *p* < 0.001). Ground-glass opacities were the most frequent radiological change at 6 and 12 months (91.7% and 87%, respectively). There was a significant reduction in the total number of lung segments with ground-glass opacities (445 to 382, *p* < 0.001) and consolidation (158 to 136, *p* = 0.019) and an increase in those with bronchiectasis (66 to 80, *p* = 0.033) between the two moments. After multivariate analysis, high-flow oxygen therapy (HFOT), highest ferritin levels, hypertension and ≥71 years showed an association with the development of subpleural parenchymal bands, consolidation, bronchiectasis and septal thickening at 6 and 12 months. *Conclusions*: Parenchymal patterns seem to be more frequent than reticular and fibrotic patterns after COVID-19 pneumonia. The fibrotic pattern was the only one that worsened significantly over time, with bronchiectasis being the only change that increased at 12 months. Older age, hypertension, the need for HFOT, and high levels of ferritin may be directly associated with worse radiological outcomes after COVID-19 pneumonia.

## 1. Introduction

According to the World Health Organization (WHO), there have been 775,583,309 declared cases of coronavirus disease 2019 (COVID-19) since the first notification in December 2019 (WHO platform consulted on 17 June 2024) [1]. The virus that causes COVID-19, severe acute respiratory syndrome coronavirus 2 (SARS-CoV-2), invades the cells through the surface receptor via angiotensin-converting enzyme 2 (ACE-2) located on type II pneumocytes. The infection can provoke a proinflammatory and prothrombotic response that manifests primarily as bilateral pneumonia, requiring hospital admission [2,3]. During the first wave of the pandemic, an estimated 10% to 20% of infected people had severe pneumonia and 20% to 25% required non-invasive respiratory support [4]. Consequently, the long-term respiratory outcomes and possible pulmonary sequelae of COVID-19, including fibrosis, have been an inevitable concern for the scientific community [5]. In this context, a recent meta-analysis indicated that after one year of COVID-19 pneumonia, up to one-third of patients have dyspnea, low diffusing capacity for carbon monoxide (DLCO) and/or radiological abnormalities [6]. Furthermore, because many of the reported radiological findings are similar to those classically associated with fibrosis or fibrosis-like changes, the course of COVID-19 pneumonia over time is of great interest [5]. The objectives of this study were to determine the main radiological findings and patterns 6 to 12 months after COVID-19 pneumonia, to explore possible risk factors for radiological abnormalities and to explore how the findings evolve between 6 and 12 months after hospital discharge.

## 2. Materials and Methods

### 2.1. Study Design and Participants

We conducted a retrospective longitudinal study in a tertiary hospital in the city of Castellón de la Plana (Spain), which has a catchment population of 283,000 inhabitants. We included consecutive adults (aged ≥ 18 years) who were admitted to the infectious diseases unit between 1 March 2020 and 30 April 2021 for COVID-19 pneumonia, confirmed via a real-time polymerase chain reaction (RT-PCR) test or an antigen test, and had a high-resolution computed tomography (HRCT) scan at 6 months and 12 months after hospital discharge. Data collection began for research purposes on 1 April 2021. Given the collapse of the healthcare system during the pandemic, the patients prioritized to receive HRCT after hospital discharge were those who needed high-flow oxygen therapy (HFOT), those who had significant radiological abnormalities at discharge, those who received biological drugs such as tocilizumab and those who were admitted to the intensive care unit (ICU).

### 2.2. Variables

We collected the following dependent variables:

- Appearance (yes/no) of radiological abnormalities at 6 and 12 months;
- Number of lung segments with radiological abnormalities at 6 and 12 months.

We grouped radiological abnormalities as follows:

- Parenchymal pattern: ground-glass opacities and consolidation;
- Reticular pattern: septal thickening, subpleural curvilinear line and subpleural parenchymal band;
- Fibrotic pattern: bronchiectasis, honeycombing and atelectasis.

These are the radiological findings most frequently associated with COVID-19 pneumonia in the literature [4,7,8,9,10,11].

To obtain our dependent variables, two thoracic radiologists independently performed a qualitative and quantitative analysis of the 6-month and 12-month HRCTs. Both radiologists were blinded to the clinical evolution of each patient. When disagreements arose, the radiologists reviewed the images together to reach a consensus.

We collected the following independent variables:

- Patient characteristics: age, sex, smoking status, cardiovascular risk factors and chronic diseases;
- Course of the disease during admission: the occurrence of acute respiratory distress syndrome (ARDS), the type of respiratory support, the minimum ratio of partial pressure arterial oxygen (PaO_2_) to the fraction of inspired oxygen (FiO_2_) and peak FiO_2_;
- ICU admission: the length of ICU stay and the length of hospital stay;
- Peak values for different laboratory parameters: C-reactive protein (CRP), ferritin, interleukin (IL)-6 and D-dimer;
- Pharmacological treatment: tocilizumab, corticosteroids and the length of corticosteroid therapy.

We extracted the independent variables from patients’ electronic medical records using the program Orion Clinic (Ministry of Universal Healthcare and Public Health, Valencian Community, Spain).

### 2.3. Statistical Analysis

We used SPSS version 23 (IBM) for all statistical analyses. First, we performed a descriptive study. For the continuous variables, we calculated means and standard deviations (SDs) if the data were normally distributed or medians and interquartile ranges (IQRs) if they followed a non-normal distribution. For categorical variables, we reported absolute and relative frequencies. Next, we studied changes in the number of affected lung segments according to each radiological finding from 6 to 12 months using the Wilcoxon signed-rank test. We also analyzed differences from 6 to 12 months in the number of patients with each type of radiological pattern using the chi-square test.

To evaluate possible associations between the independent and dependent variables at 6 and 12 months, we calculated odds ratios (ORs) with 95% confidence intervals (CIs). To do this, we dichotomized the continuous variables using cut-off points that represented the 75th percentile in each case. Lastly, we fitted a binary logistic regression model, including the independent variables that had shown a statistically significant association with any dependent variable in the univariate analysis, and adjusted for sex and age. In the multivariate analysis, we only included variables that were available for the whole sample; for this reason, we excluded peak IL-6, nadir PaO_2_/FiO_2_ and the duration of ICU stay. We considered *p* values below 0.050 representative of statistical significance.

### 2.4. Ethical Aspects

The Research Ethics Committee of Castellón General University Hospital approved this study according to the guidelines of the Spanish Agency for Medicines and Medical Devices (AEMPS). The Medicine Research Ethics Committee of Castellón General University Hospital approved the waiver of informed consent, and data were obtained via an anonymized form. We ensured patient confidentiality and data protection in accordance with Regulation (EU) 2016/679 of the European Parliament and of the Council of 27 April 2016 on the protection of natural persons with regard to the processing of personal data and on the free movement of such data.

## 3. Results

### 3.1. Study Sample

Table 1 presents the results of the descriptive analysis.

Our sample comprised 108 patients with a mean age of 64 years (SD 9). Most patients were men (n = 77, 71.3%). The main comorbidities were hypertension (n = 52, 48.1%) and obesity (n = 43, 39.8%). No patients had received any doses of a SARS-CoV-2 vaccine. More than two-thirds of patients had ARDS (n = 75, 69.4%), and more than half were admitted to the ICU (n = 63, 58.3%). The main type of respiratory support was helmet continuous positive airway pressure (CPAP; n = 59, 54.6%). Eight patients (7.4%) required invasive mechanical ventilation.

The main type of pharmacological treatment was corticosteroid therapy, administered to 105 patients (97.2%) for a median duration of 54 days (IQR 41–81). Only 16 patients (14.8%) received tocilizumab. The median peak values for blood test parameters during admission were as follows: CRP: 118.5 mg/L (IQR 62.2–193.7), ferritin: 851 μg/L (IQR 534.2–1728), IL-6: 59.1 ng/L (IQR 24.9–138) and D-dimer: 2005 ng/mL (IQR 912.5–4175).

### 3.2. Changes in the Main Radiological Patterns and Findings from 6 to 12 Months After Hospital Discharge

Table 2 presents the data on radiological patterns and findings at 6 and 12 months.

#### 3.2.1. Radiological Patterns

**Figure 1:**
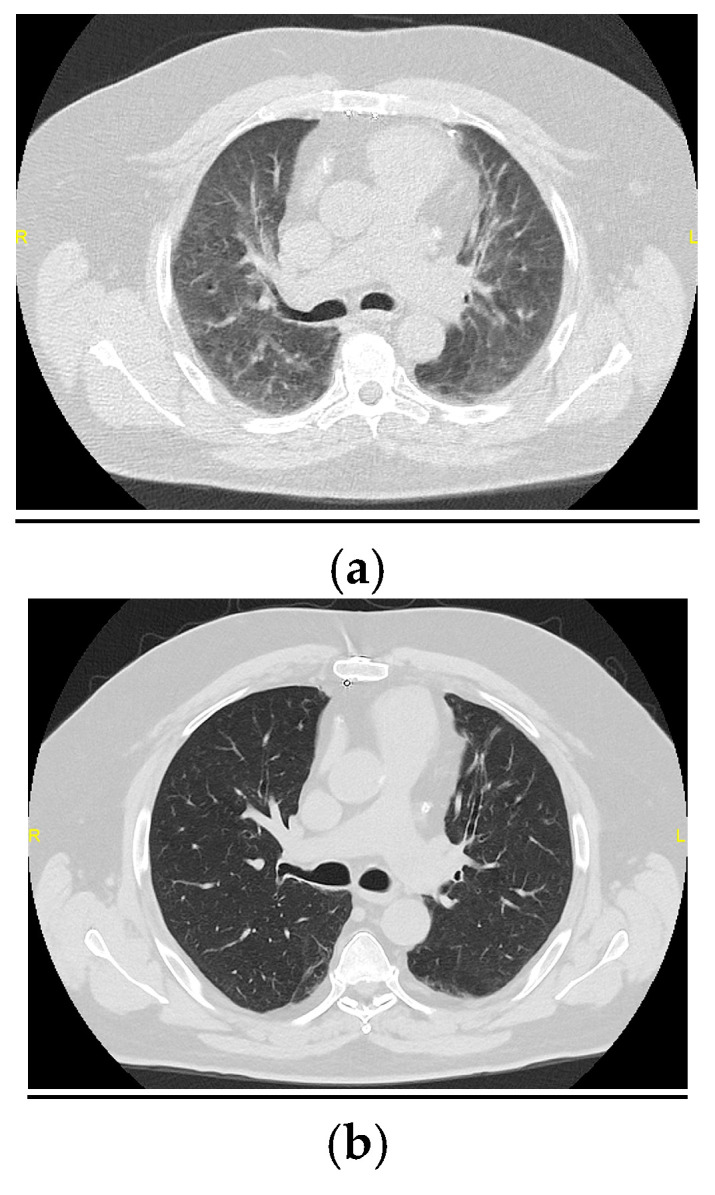
(**a**) HRCT image performed 6 months after hospital discharge showing diffuse ground-glass opacities and cylindrical bronchiectasis. (**b**) Image demonstrating complete resolution of the ground-glass opacities 12 months after hospital discharge, with the persistence of the bronchiectasis.

**Figure 2:**
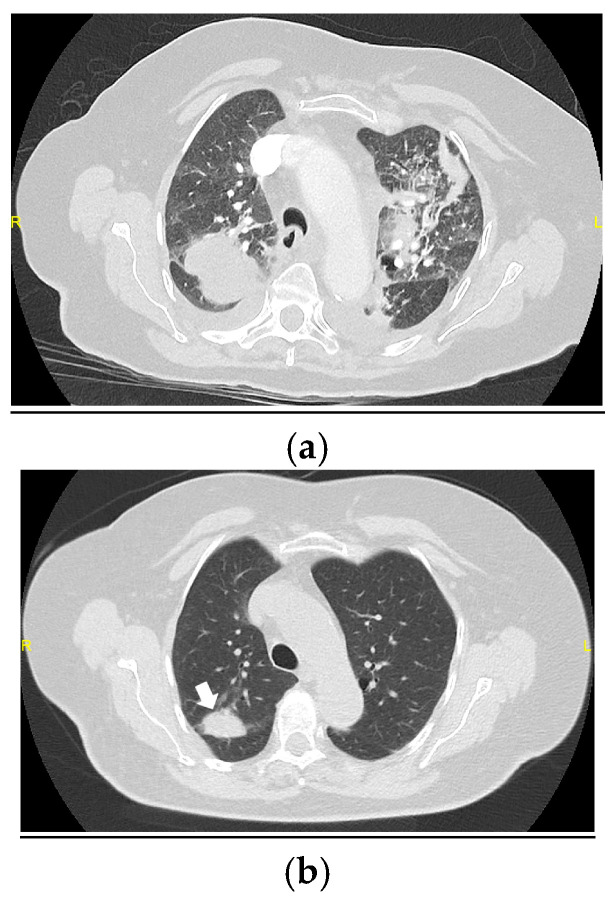
(**a**) HRCT performed 6 months after hospital discharge, showing diffuse ground-glass opacities, multilobar parenchymal consolidations with a predominantly central distribution and bilateral pleural effusion. (**b**) HRCT at 12 months, demonstrating the near-complete resolution of pulmonary abnormalities, except for a small persistent consolidation in the right upper lobe (arrow).

**Figure 3:**
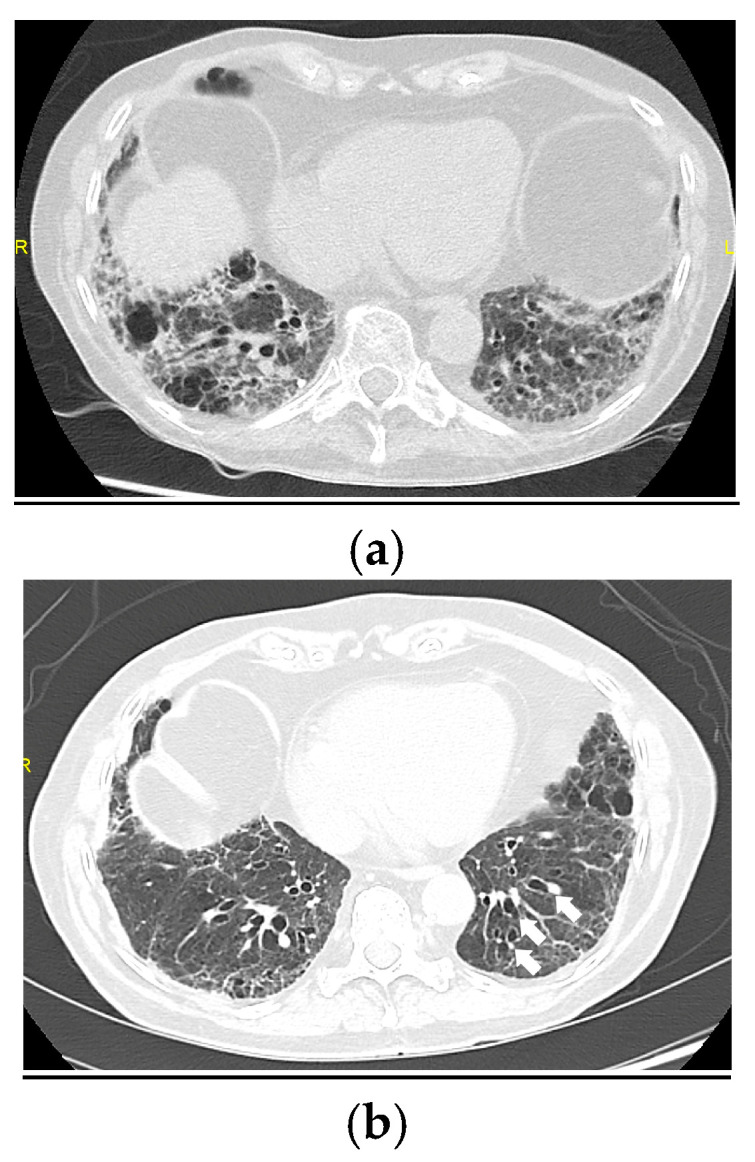
(**a**) Follow-up HRCT at 6 months after hospital discharge. Image showing ground-glass opacities and septal thickening in both lower lobes, with cylindrical bronchiectasis in the right lower lobe. (**b**) Follow-up image at 12 months showing a radiological improvement of the ground-glass opacities, but with the development or worsening of bronchiectasis in the left lower lobe (arrows); the findings are suggestive of post-COVID-19 sequelae.

From 6 months to 12 months of follow-up, we observed a statistically significant decrease in the proportion of people with parenchymal patterns (from 93.5% to 88.9%, *p* < 0.001) and reticular patterns (from 63% to 62%, *p* < 0.001). There was a significant increase in the proportion with fibrotic patterns (from 62% to 63.9%, *p* < 0.001). In addition, we observed an improvement from 6 months to 12 months in the number of lung segments with parenchymal lesions in 38.9% of the sample (Figure 1 and Figure 2). For reticular lesions, this proportion was 14.8%. However, only 10.2% of patients had fewer lung segments with fibrotic lesions at 12 months compared to 6 months, and 16.7% had more lung segments with fibrotic lesions (Figure 3).

#### 3.2.2. Main Radiological Findings

At six months, the most common radiological findings were ground-glass opacities (n = 99, 91.7%), consolidation (n = 57, 52.8%) and subpleural parenchymal bands (n = 42, 38.9%). The predominant radiological findings were similar at 12 months: ground-glass opacities (n = 94, 87%), consolidation (n = 53, 49.1%) and subpleural parenchymal bands (n = 43, 39.8%). We found a statistically significant reduction, derived via the Wilcoxon signed-rank test, from 6 to 12 months in the number of lung segments with ground-glass opacities (total of lung segments affected of 445 to 382, *p* < 0.001) and consolidation (total of lung segments affected of 158 to 136, *p* = 0.019) and an increase in the number of lung segments with bronchiectasis (total of lung segments affected of 66 to 80, *p* = 0.033).

### 3.3. Risk Factors for the Main Radiological Findings at 6 and 12 Months After Hospital Discharge

We performed a univariate analysis of possible risk factors related to the radiological findings observed at 6 months and 12 months (Table 3 and Table 4).

Subsequently, after adjusting for sex and age, we fitted a multivariate model with all variables that showed statistical significance in the univariate analysis (Table 5 and Table 6).

We found associations between HFOT and the presence of subpleural parenchymal bands at 6 months (aOR 10.032, 95% CI 1.133 to 88.807) and 12 months (aOR 12.612, 95% CI 1.483 to 107.287), between peak ferritin levels of 1728 μg/L or greater and the presence of consolidation (aOR 3.291, 95% CI 1.246 to 8.697) and bronchiectasis at 12 months (aOR 3.140, 95% CI 1.150 to 8.576), between hypertension and septal thickening at 12 months (aOR 2.968, 95% CI 1.020 to 8.637) and between ages over 70 years and septal thickening at 12 months (aOR 3.554, 95% CI 1.156 to 10.929).

## 4. Discussion

According to our results, the predominant radiological pattern during the first year after COVID-19 pneumonia is parenchymal; the incidence of reticular and fibrotic patterns was much lower. In a systematic review published in 2022, Watanabe and colleagues synthesized 15 studies of radiological sequelae over one year in 1801 people with COVID-19 pneumonia, finding a similar predominance of parenchymal involvement [6]. However, a larger proportion of patients in our population had radiological abnormalities of any type. For example, 12 months after hospital discharge, 87% of our sample had ground-glass opacities and 38.9% had bronchiectasis, compared with 21.2% and 9%, respectively, as described in the meta-analysis by Watanabe et al. [6]. In contrast, a recent Spanish study found that the reticular pattern (31.9%) and the fibrotic pattern (27.3%) were more frequent than the parenchymal pattern one year after pneumonia [12]. In short, radiological abnormalities are very frequent one year after COVID-19 pneumonia, but there is considerable heterogeneity in the results, probably due to several factors such as differences in the populations analyzed or even inter-observer variability between the radiologists who interpreted the images. In our study, two thoracic radiologists independently reviewed all HRCT images, which surely increased the sensitivity of the analysis.

We noticed significant improvements over time in the parenchymal and reticular patterns in terms of the absolute number of affected patients and the median number of affected lung regions. In contrast, the fibrosis-like abnormalities worsened between 6 and 12 months of follow-up, with a significant increase in the number of patients with bronchiectasis and the median number of lung segments with bronchiectasis. Eizaguirre and colleagues presented similar findings with regard to fibrotic-like abnormalities [12]. However, other studies have described improvements over time in fibrotic abnormalities, even establishing the hypothesis that this finding reflects the remodeling of “immature” fibrosis [10,11,13,14].

The literature contains less evidence on the risk factors that influence radiological outcomes after COVID-19 pneumonia. We found that older age, a history of hypertension, a need for HFOT and high ferritin levels during hospital stay may be risk factors for the main radiological lung lesions. Other studies have proposed age and the severity of pneumonia as predictors of worse long-term radiological outcomes [4,15,16,17]. In fact, a previous study carried out by our group that analyzed the first 189 who had an HRCT 6 months after hospital discharge also pointed out age as the main risk factor for practically all radiological changes, in addition to a longer hospital stay in an ICU or elevated levels of IL-6 [18]. Of the various methods of respiratory support (such as HFOT, non-invasive mechanical ventilation with helmet CPAP and invasive mechanical ventilation), only HFOT was associated with radiological lesions at 6 and 12 months in our study, probably because it requires high FiO_2_ levels. Using methods that enable greater alveolar recruitment, such as CPAP, could help mitigate this increased risk [19]. Furthermore, research has shown associations between greater inflammatory response and greater pneumonia severity (and thus greater lung damage), which could explain the influence of the high ferritin levels on the appearance of different radiological findings in our population [20,21].

In contrast, no benefit was observed with the use of systemic corticosteroids during hospital admission, nor was the duration of treatment influential. It should be noted that given the high frequency of prolonged persistence of interstitial lung disease and organizing pneumonia after COVID-19 pneumonia in our hospital, we proposed prolonging corticosteroid therapy for around 6 weeks in descending doses. Expert opinions and subsequent case series support this decision [22].

The aim of our data collection process for radiological variables (independent review of each case by two radiologists, followed by a consensus process wherever disagreements arose) was to ensure an accurate and reliable description of lung damage and to minimize inter-observer variability. Furthermore, since nobody in our population had been vaccinated against COVID-19, we avoided the mitigating impact this could have had on radiological outcomes.

However, this study also has several limitations. First, it has a retrospective design and a sample size similar to other studies that have mostly used non-parametric statistical tests. We had a limited number of participants because people with lung disease are frequently admitted to the pulmonology department in our hospital. Second, we were unable to evaluate the impact of antiviral treatments because they were not available in our hospital. Third, the administration or non-administration of antibiotic therapy during hospitalization or the outpatient use of other anti-inflammatory drugs has not been analyzed. Finally, we had to exclude some patients from the multivariate analysis because they had missing values for one or more continuous variables.

## 5. Conclusions

Our results suggest that long-term radiological abnormalities after COVID-19 pneumonia were common even one year after COVID-19 pneumonia. Parenchymal patterns seem to be more frequent than reticular and fibrotic patterns, and the fibrotic pattern was the only one that worsened significantly over time. This was evidenced by a significant increase in the number of lung segments with bronchiectasis at 12 months, while other parenchymal changes such as ground-glass opacities or consolidation improved. Older age, hypertension, the need for HFOT, and excessive inflammation represented by high levels of ferritin may be directly associated with worse radiological outcomes, though larger studies with greater statistical power are needed to confirm these findings. Our results could be helpful for predicting or preventing certain radiological outcomes in the long term.

## Figures and Tables

**Table 1:** Descriptive study of the sample (n = 108) ^a^.

Categorical Variables	n (%)
General characteristics	
Male	77 (71.3)
Smoker/ex-smoker	36 (33.3)
Comorbidities	
Hypertension	52 (48.1)
Dyslipidemia	35 (32.4)
Obesity (BMI > 30 km/m^2^)	43 (39.8)
Ischemic heart disease	6 (5.6)
Heart failure	3 (2.8)
Chronic bronchopathy	2 (1.8)
Chronic kidney disease	2 (1.8)
Diabetes	16 (14.8)
Disease course	
ARDS	75 (69.4)
ICU admission	63 (58.3)
Helmet CPAP	59 (54.6)
High-flow oxygen therapy	8 (7.4)
Invasive mechanical ventilation	8 (7.4)
Pharmacological treatment	
Tocilizumab	16 (14.8)
Corticosteroids	105 (97.2)
Continuous variables	Median (IQR) ^b^
Age (years), mean (SD)	64 (9)
Laboratory values	
Peak CRP (mg/L)	118.5 (62.2–193.7)
Peak ferritin (µg/L)	851 (534.2–1728)
Peak IL-6 (ng/L) [n = 96]	59.1 (24.9–138)
Peak D-dimer (ng/mL)	2005 (912.5–4175)
Nadir PaO_2_/FiO_2_ (%) [n = 105],	166 (105.5–312)
Peak FiO_2_ (%)	60 (32–60)
Duration of steroid therapy (days)	54 (41–81)
Duration of ICU stay (days) [n = 63]	7 (4–10)
Duration of hospital stay (days)	15 (9–22)

^a^ n (total number of participants) was 108, unless otherwise specified in square brackets in the variables column. ^b^ Except for age, which is expressed as the mean (SD), all other variables are shown as the median (IQR). Abbreviations: ARDS, acute respiratory distress syndrome; BMI, body mass index; CPAP: continuous positive airway pressure; CRP, C-reactive protein; FiO_2_, fraction of inspired oxygen; ICU: intensive care unit; IL-6, interleukin 6; IQR, interquartile range; PaO_2_, partial pressure of oxygen; SD, standard deviation.

**Table 2:** High-resolution computer tomography findings 6 and 12 months after COVID-19 pneumonia.

	No. Lung Segments Affected, Total		No. Lung Segments Affected, Mdn (IQR)			Change from 6 to 12 Months, n (%) ^a^		
	6 Months	12 Months	6 Months	12 Months	*p* Value ^b^	Improvement	Worsening	Stability
**Parenchymal pattern**			6 (3.2–8)	5 (2–6)	<0.001	42 (38.9)	3 (2.8)	63 (58.3)
Ground-glass opacities	445	382	5 (2–6)	3.5 (2–6)	<0.001	35 (32.4)	0	73 (67.6)
Consolidation	158	136	1 (0–2)	0 (0–2)	0.019	19 (17.6)	7 (6.5)	82 (75.9)
**Reticular pattern**			1 (0–3)	1 (0–2)	0.171	16 (14.8)	10 (9.3)	82 (75.9)
Septal thickening	35	28	0 (0–1)	0 (0–1)	0.166	7 (6.5)	3 (2.8)	98 (90.7)
Subpleural curvilinear line	41	40	0 (0–1)	0 (0–1)	0.705	4 (3.7)	3 (2.8)	101 (93.5)
Subpleural parenchymal bands	101	97	0 (0–2)	0 (0–2)	0.612	8 (7.4)	7 (6.5)	93 (86.1)
**Fibrotic pattern**			1 (0–2)	1 (0–2)	0.186	11 (10.2)	18 (16.7)	79 (73.1)
Bronchiectasis	66	80	0 (0–1)	0 (0–1)	0.033	5 (4.6)	14 (13)	89 (82.4)
Honeycombing	20	22	0 (0–0)	0 (0–0)	0.317	0	1 (0.9)	107 (99.1)
Atelectasis	60	54	0 (0–1)	0 (0–1)	0.222	8 (7.4)	4 (3.7)	96 (88.9)
			**n (%)**	**n (%)**	***p* value ^c^**	—	—	—
**Parenchymal pattern**			101 (93.5)	96 (88.9)	<0.001	—	—	—
**Reticular pattern**			68 (63)	67 (62)	<0.001	—	—	—
**Fibrotic pattern**			67 (62)	69 (63.9)	<0.001	—	—	—

^a^ Radiological improvement, worsening or stability is defined by the decrease, increase or stability in the number of affected lung regions. ^b^ Obtained using the Wilcoxon signed-rank test. ^c^ Obtained via χ^2^. Abbreviations: IQR: interquartile range; Mdn: median.

**Table 3:** Risk factors for the occurrence of radiological abnormalities six months after COVID-19 pneumonia.

	Ground-Glass Opacities, n = 99 (91.7%)		Consolidation, n = 57 (52.8%)		Septal Thickening, n = 32 (29.6%)		Subpleural Curvilinear Line, n = 28 (25.9%)		Subpleural Parenchymal Bands, n = 42 (38.9%)		Bronchiectasis, n = 38 (35.2%)		Honeycombing, n = 5 (4.6%)		Atelectasis, n = 38 (35.5%)	
	OR	95% CI	OR	95% CI	OR	95% CI	OR	95% CI	OR	95% CI	OR	95% CI	OR	95% CI	OR	95% CI
**General characteristics**																
Male	2.133	0.533–8.541	0.741	0.319–1.720	0.840	0.341.2.068	0.515	0.207–1.282	1.225	0.516–2.911	2.311	0.887–6.018	1.644	0.176–15.320	1.200	0.495–2.907
Smoker/ex-smoker	0.597	0.150–2.374	0.510	0.227–1.149	0.571	0.226–1.443	0.455	0.166–1.248	0.481	0.202–1.142	2.173	0.949–4.976	3.182	0.507–19.963	1.522	0.665–3.480
**Comorbidities**																
Hypertension	1.176	0.298–4.642	1.465	0.685–3.131	2.292	0.981–5.352	0.911	0.385–2.158	0.966	0.445–2.095	0.810	0.366–1.789	1.653	0.265–10.310	0.953	0.432–2.101
Dyslipidemia	0.570	0.143–2.269	0.923	0.412–2.068	2.037	0.862–4.817	0.984	0.392–2.471	2.162	0.949–4.925	0.643	0.269–1.537	3.328	0.530–20.897	1.361	0.591–3.134
Obesity (BMI > 30 km/m^2^)	1.356	0.320–5.739	1.225	0.565–2.655	1.815	0.786–4.192	1.444	0.605–3.447	0.889	0.402–1.964	0.693	0.305–1.575	2.363	0.378–14.765	1.370	0.614–3.056
Ischemic heart disease	1.065	1.013–1.119	4.808	0.542–42.609	1.200	0.209–6.906	1.462	0.253–8.451	0.775	0.136–4.430	1.914	0.367–9.984	4.900	0.459–52.333	4.000	0.697–22.940
Heart failure	1.031	0.996–1.068	1.818	0.160–20.669	0.961	0.918–1.005	6.077	0.529–69.792	0.780	0.069–8.886	0.957	0.911–1.006	0.971	0.939–1.004	1.086	0.989–1.192
Chronic bronchopathy	1.021	0.992–1.050	1.036	0.986–1.089	2.419	0.147–39.914	0.975	0.941–1.010	1.585	0.096–26.051	1.865	0.113–30.680	0.981	0.954–1.008	1.865	0.113–30.680
Chronic kidney disease	1.021	0.992–1.050	0.893	0.054–14.652	2.419	0.147–39.914	0.975	0.941–1.010	1.585	0.096–26.051	1.865	0.113–30.680	0.981	0.954–1.008	0.971	0.933–1.011
Diabetes	0.302	0.067–1.360	0.653	0.224–1.904	1.094	0.347–3.451	0.944	0.278–3.210	1.706	0.587–4.961	0.813	0.260–2.540	1.467	0.153–14.036	0.813	0.260–2.540
**Disease course**																
ARDS	5.333	1.245–22.845	1.281	0.564–2.910	3.150	1.089–9.110	0.905	0.358–2.284	0.809	0.351–1.862	1.370	0.570–3.295	0.646	0.103–4.059	0.929	0.395–2.181
ICU admission	13.405	1.612–111.499	0.963	0.447–2.071	1.545	0.655–3.646	0.769	0.323–1.831	0.923	0.422–2.021	1.619	0.714–3.672	0.459	0.073–2.867	1.150	0.514–2.571
Helmet CPAP	11.317	1.363–93.999	0.843	0.394–1.802	1.581	0.679–3.682	0.944	0.398–2.239	0.629	0.289–1.372	1.226	0.553–2.721	0.538	0.086–3.357	1.226	0.553–2.721
High-flow oxygen therapy	1.088	1.026–1.153	0.887	0.210–3.745	0.778	0.148–4.076	0.949	0.180–4.997	13.000	1.537–109.964	1.114	0.251–4.940	0.922	0.872–0.975	0.593	0.114–3.090
Invasive mechanical ventilation	1.088	1.026–1.153	7.000	0.830–59.001	4.506	1.008–20.153	0.900	0.837–0.968	5.333	1.023–27.814	6.375	1.219–33.346	0.922	0.872–0.975	0.886	0.814–0.963
**Treatment**																
Tocilizumab	1.193	1.094–1.301	1.596	0.536–4.754	1.523	0.502–4.618	0.363	0.077–1.708	1.267	0.433–3.706	2.067	0.707–6.041	1.467	0.153–14.036	1.530	0.521–4.499
Corticosteroids	1.031	0.996–1.068	1.056	0.993–1.122	0.200	0.017–2.289	0.963	0.922–1.005	1.077	0.990–1.171	0.261	0.023–2.975	0.971	0.939–1.004	0.957	0.911–1.006
**Dichotomized continuous variables**																
Age ≥ 71 years	0.538	0.124–2.336	1.335	0.533–3.341	1.591	0.612–4.137	1.600	0.597–4.289	1.161	0.461–2.922	1.138	0.444–2.916	2.455	0.386–15.614	0.900	0.345–2.348
Peak CRP ≥ 193.7 mg/L	2.849	0.340–23.893	1.743	0.712–4.262	3.030	1.216–7.552	1.000	0.370–2.702	1.109	0.456–2.697	2.080	0.854–5.065	0.738	0.658–0.828	0.565	0.214–1.490
Peak ferritin ≥ 1728 µg/L	2.849	0.340–23.893	2.154	0.866–5.357	1.000	0.385–2.596	0.573	0.194–1.695	2.038	0.843–4.929	2.558	1.047–6.247	0.738	0.658–0.828	0.897	0.357–2.250
Peak IL-6 ≥ 138 ng/L	1.369	1.207–1.553	1.667	0.647–4.296	1.500	0.550–4.087	0.325	0.088–1.203	1.410	0.554–3.589	1.803	0.702–4.631	0.739	0.079–6.955	0.590	0.208–1.671
Peak D-dimer ≥ 4175 ng/mL	0.640	0.149–2.756	0.952	0.398–2-276	3.030	1.216–7.552	0.413	0.129–1.323	2.038	0.843–4.929	2.080	0.854–5.065	2.080	0.329–13.163	0.761	0.279–1.834
Nadir PaO_2_/FiO_2_ ≤ 312%	4.464	1.100–18.122	1.394	0.573–3.390	3.981	1.096–14.459	0.920	0.337–2.511	0.980	0.394–2.437	1.574	0.591–4.194	0.474	0.075–3.004	0.878	0.352–2.193
Peak FiO_2_ ≥ 60%	1.303	1.169–1.452	1.519	0.594–3.886	1.050	0.385–2.864	0.749	0.249–2.250	1.013	0.394–2.604	1.969	0.772–5.026	0.920	0.098–8.658	0.585	0.209–1.636
Duration of steroid therapy ≥ 80 days	1.182	0.230–6.069	1.161	0.484–2.785	1.578	0.628–3.966	2.059	0.804–5.273	1.109	0.456–2.697	1.113	0.450–2.754	2.080	0.329–13.163	1.375	0.561–3.368
ICU stay ≥ 10 days	5.636	3.298–9.633	0.889	0.253–3.128	1.563	0.430–5.682	0.240	0.028–2.037	2.800	0.773–10.140	6.563	1.564–27.540	4.545	0.264–78.394	0.286	0.057–1.440
Hospital stay ≥ 22 days	2.286	0.271–19.276	1.519	0.594–3.886	1.733	0.661–4.546	0.749	0.249–2.250	1.274	0.501–3.242	2.476	0.968–6.333	0.920	0.098–8.658	1.241	0.480–3.209

Abbreviations: ARDS, acute respiratory distress syndrome; BMI, body mass index; CI, confidence interval; CPAP, continuous positive airway pressure; CRP, C-reactive protein; FiO_2_, fraction of inspired oxygen; ICU, intensive care unit; IL-6, interleukin 6; IQR, interquartile range; OR, odds ratio; PaO_2_, partial pressure of oxygen; SD, standard deviation.

**Table 4:** Risk factors for the occurrence of radiological abnormalities 12 months after COVID-19 pneumonia.

	Ground-Glass Opacities, n = 94 (87%)		Consolidation, n = 53 (49.1%)		Septal Thickening, n = 28 (25.9%)		Subpleural Curvilinear Line, n = 29 (26.9%)		Subpleural Parenchymal Bands, n = 43 (39.8%)		Bronchiectasis, n = 42 (38.9%)		Honeycombing, n = 6 (5.6%)		Atelectasis, n = 36 (33.3%)	
	OR	95% CI	OR	95% CI	OR	95% CI	OR	95% CI	OR	95% CI	OR	95% CI	OR	95% CI	OR	95% CI
**General characteristics**																
Male	0.993	0.286–3.439	0.867	0.377–1.997	1.286	0.483–3.426	0.688	0.276–1.716	0.884	0.379–2.063	2.273	0.904–5.714	2.083	0.233–18.594	1.643	0.649–4.159
Smoker/ex-smoker	0.318	0.101–1.002	0.757	0.339–1.690	0.930	0.371–2.330	0.426	0.156–1.166	0.455	0.192–1.079	2.000	0.884–4.526	4.375	0.762–25.130	1.446	0.626–3.340
**Comorbidities**																
Hypertension	0.660	0.212–2.050	1.447	0.678–3.088	3.750	1.474–9.542	1.007	0.430–2.360	0.768	0.354–1.664	0.602	0.275–1.318	1.082	0.208–5.614	1.321	0.592–2.945
Dyslipidemia	0.424	0.136–1.323	0.691	0.307–1.556	2.285	0.938–5.565	0.917	0.367–2.293	1.435	0.634–3.249	0.897	0.391–2.057	2.188	0.418–11.437	1.285	0.552–2.990
Obesity (BMI > 30 km/m^2^)	0.621	0.201–1.916	1.342	0.620–2.905	1.759	0.737–4.197	1.609	0.681–3.803	0.708	0.319–1.570	0.889	0.402–1.964	3.231	0.565–18.475	0.944	0.416–2.140
Ischemic heart disease	0.267	0.044–1.615	1.040	0.200–5.398	1.462	0.253–8.451	0.529	0.059–4.726	0.286	0.032–2.535	3.368	0.589–19.272	3.880	0.378–39.782	2.091	0.400–10.922
Heart failure	1.033	0.996–1.072	2.118	0.186–24.073	1.444	0.126–16.573	5.778	0.504–66.290	0.750	0.066–8.536	0.780	0.069–8.886	0.971	0.938–1.004	1.091	0.989–1.204
Chronic bronchopathy	1.022	0.992–1.053	1.039	0.985–1.096	1.077	0.972–1.193	0.975	0.941–1.010	1.524	0.093–25.033	1.050	0.981–1.123	0.980	0.954–1.008	2.029	0.123–33.400
Chronic kidney disease	1.022	0.992–1.053	1.038	0.063–17.040	2.926	0.177–48.407	0.975	0.941–1.010	1.524	0.093–25.033	1.585	0.096–26.051	0.980	0.954–1.008	0.972	0.935–1.011
Diabetes	0.366	0.099–1.354	1.044	0.361–3.020	2.630	0.874–7.909	1.288	0.406–4.088	1.629	0.561–4.732	0.676	0.217–2.105	1.160	0.127–10.636	0.894	0.285–2.803
**Disease course**																
ARDS	2.615	0.836–8.186	1.035	0.456–2.347	1.868	0.677–5.153	0.970	0.386–2.436	0.598	0.261–1.370	0.970	0.419–2.244	0.873	0.152–5.020	0.680	0.290–1.598
ICU admission	2.900	0.900–9.340	0.870	0.404–1.870	1.142	0.475–2.750	0.692	0.294–1.630	0.719	0.329–1.568	1.084	0.494–2.378	0.700	0.135–3.638	0.843	0.376–1.893
Helmet CPAP	2.430	0.756–7.807	0.746	0.349–1.595	0.944	0.398–2.239	0.852	0.363–2.000	0.580	0.266–1.262	0.862	0.397–1.874	0.821	0.158–4.265	0.894	0.401–1.995
High-flow oxygen therapy	1.093	1.028–1.163	1.806	0.409–7.965	1.800	0.401–8.077	3.000	0.698–12.891	12.444	1.472–105.212	1.632	0.385–6.911	0.922	0.871–0.975	0.889	0.819–0.965
Invasive mechanical ventilation	1.093	1.028–1.163	8.217	0.975–69.278	10.636	2.005–56.433	0.367	0.043–3.123	2.719	0.615–12.032	5.333	1.023–27.814	0.922	0.871–0.975	0.889	0.819–0.965
**Treatment**																
Tocilizumab	2.468	0.300–20.309	1.403	0.482–4.085	1.909	0.623–5.850	0.344	0.073–1.617	1.210	0.414–3.537	1.267	0.433–3.706	1.160	0.127–10.636	1.690	0.573–4.982
Corticosteroids	1.033	0.996–1.072	0.472	0.042–5.368	0.165	0.014–1.890	0.962	0.921–1.005	0.320	0.028–3.647	0.308	0.027–3.504	0.971	0.938–1.004	0.958	0.913–1.006
**Dichotomized continuous variables**																
Age ≥ 71 years	1.055	0.269–4.133	2.018	0.795–5.121	3.335	1.273–8.739	1.920	0.730–5.050	1.710	0.685–4.267	1.800	0.720–4.500	1.818	0.312–10.586	1.267	0.492–3.258
Peak CRP ≥ 193.7 mg/L	4.971	0.619–39.929	1.414	0.589–3.395	2.588	1.016–6.597	0.939	0.349–2.527	1.053	0.433–2.557	1.665	0.690–4.018	0.735	0.654–0.826	0.367	0.126–1.068
Peak ferritin ≥ 1728 µg/L	4.971	0.619–39.929	2.629	1.055–6.550	1.632	0.630–4.223	0.720	0.258–2.014	1.579	0.655–3.803	3.808	1.529–9.482	0.735	0.654–0.826	1.000	0.397–2.519
Peak IL-6 ≥ 138 ng/L	3.710	0.449–30.616	1.750	0.687–4.459	2.723	0.980–7.563	0.639	0.211–1.936	1.410	0.554–3.589	1.410	0.554–3.589	0.583	0.065–5.251	0.667	0.234–1.897
Peak D-dimer ≥ 4175 ng/mL	1.257	0.323–4.889	1.160	0.485–2.773	5.154	1.994–13.320	0.540	0.183–1.592	1.290	0.534–3.115	1.360	0.562–3.288	1.540	0.266–8.918	0.628	0.237–1.661
Nadir PaO_2_/FiO_2_ ≤ 312%	2.663	0.827–8.570	1.138	0.468–2.765	2.123	0.657–6.863	0.983	0.362–2.672	0.677	0.276–1.659	0.980	0.394–2.437	0.640	0.110–3.714	0.631	0.254–1.570
Peak FiO_2_ ≥ 60%	1.726	0.358–8.327	1.463	0.578–3.699	1.333	0.483–3.682	0.706	0.236–2.116	0.964	0.375–2.476	2.000	0.788–5.075	0.727	0.081–6.552	0.647	0.231–1.815
Duration of steroid therapy ≥ 80 days	2.174	0.454–10.400	1.730	0.715–4.185	1.632	0.630–4.223	1.920	0.754–4.889	1.579	0.655–3.803	1.360	0.562–3.288	1.540	0.266–8.918	0.628	0.237–1.661
ICU stay ≥ 10 days	0.936	0.095–9.222	0.743	0.208–2.651	2.321	0.622–8.668	0.585	0.113–3.025	2.000	0.560–7.140	6.563	1.564–27.540	2.227	0.185–26.808	0.721	0.599–0.868
Hospital stay ≥ 22 days	3.972	0.492–32.091	1.835	0.717–4.696	2.863	1.080–7.590	0.706	0.236–2.116	1.212	0.477–3.080	2.000	0.788–5.075	0.727	0.081–6.552	1.086	0.412–2.864

Abbreviations: ARDS, acute respiratory distress syndrome; BMI, body mass index; CI, confidence interval; CPAP, continuous positive airway pressure; CRP, C-reactive protein; FiO_2_, fraction of inspired oxygen; ICU, intensive care unit; IL-6, interleukin 6; IQR, interquartile range; OR, odds ratio; PaO_2_, partial pressure of oxygen; SD, standard deviation.

**Table 5:** Multivariate analysis of risk factors for major radiological abnormalities at six months after COVID-19 pneumonia.

	**Ground-Glass Opacities**			**Consolidation**			**Septal Thickening**			**Subpleural Curvilinear Line**		
	**aOR**	**95% CI**	***p***	**aOR**	**95% CI**	***p***	**aOR**	**95% CI**	***p***	**aOR**	**95% CI**	***p***
Male	1.539	0.352–6.725	0.567	0.751	0.323–1.746	0.505	0.638	0.235–1.731	0.378	0.569	0.225–1.440	0.234
ARDS	0.881	0.145–5.358	0.891	—	—	—	2.995	0.928–9.666	0.066	—	—	—
Invasive mechanical ventilation	9,297,672	0.000–NA	0.999	—	—	—	1.567	0.283–8.678	0.607	0.000	0.000–NA	0.999
Age ≥ 71 years	0.979	0.203–4.731	0.979	1.314	0.523–3.298	0.561	2.301	0.776–6.819	0.133	1.592	0.576–4.399	0.370
Peak CRP ≥ 193.7 mg/L	—	—	—	—	—	—	2.281	0.800–6.500	0.123	—	—	—
Max D-dimer ≥ 4175 ng/mL	—	—	—	—	—	—	1.918	0.668–5.507	0.226	—	—	—
	**Subpleural Parenchymal Bands**			**Bronchiectasis**			**Honeycombing**			**Atelectasis**		
	**aOR**	**95% CI**	***p***	**aOR**	**95% CI**	***p***	**aOR**	**95% CI**	***p***	**aOR**	**95% CI**	***p***
Male	1.166	0.470–2.829	0.740	1.905	0.695–5.224	0.211	2.347	0.243–22.662	0.461	1.327	0.540–3.263	0.537
High flow oxygen therapy	10.032	1.133–88.807	0.038	—	—	—	0.000	0.000–NA	0.999	—	—	—
Invasive mechanical ventilation	3.368	0.571–19.873	0.180	4.473	0.793–25.234	0.090	1.580	0.000–NA	1.000	0.000	0.000–NA	0.999
Age ≥ 71 years	1.161	0.437–3.086	0.765	1.259	0.462–3.429	0.653	2.167	0.324–14.509	0.425	0.935	0.349–2.506	0.894
Peak ferritin ≥ 1728 µg/L	—	—	—	1.722	0.639–4.644	0.283	0.000	0.000–NA	0.998	—	—	—

Abbreviations: aOR, adjusted odds ratio; ARDS, acute respiratory distress syndrome; CI, confidence interval; CPAP, continuous positive airway pressure; CRP, C-reactive protein; FiO_2_, fraction of inspired oxygen; ICU, intensive care unit; NA, not available.

**Table 6:** Multivariate analysis of risk factors for radiological abnormalities at 12 months after COVID-19 pneumonia.

	**Ground-Glass Opacities**			**Consolidation**			**Septal Thickening**			**Subpleural Curvilinear Line**		
	**aOR**	**95% CI**	***p***	**aOR**	**95% CI**	***p***	**aOR**	**95% CI**	***p***	**aOR**	**95% CI**	***p***
Male	0.954	0.270–3.362	0.941	0.660	0.270–1.614	0.362	0.947	0.288–3.119	0.929	0.708	0.281–1.780	0.462
Hypertension	—	—	—	—	—	—	2.968	1.020–8.637	0.046	—	—	—
High flow oxygen therapy	151,040,397	0.000–NA	0.999	—	—	—	—	—	—	—	—	—
Mechanical ventilation	152,483,294	0.000–NA	0.999	—	—	—	2.492	0.342–18.150	0.367	—	—	—
Age ≥ 71 years	1.042	0.260–4.171	0.953	2.308	0.882–6.037	0.088	3.554	1.156–10.929	0.027	1.887	0.715–4.980	0.200
Peak CRP ≥ 193.7 mg/L	—	—	—	—	—	—	2.343	0.682–8.044	0.176	—	—	—
Peak ferritin ≥ 1728 µg/L	—	—	—	3.291	1.246–8.697	0.016	—	—	—	—	—	—
Peak D-dimer ≥ 4175 ng/mL	—	—	—	—	—	—	3.023	0.975–9.374	0.055	—	—	—
Hospital stay ≥ 22 days	—	—	—	—	—	—	1.651	0.496–5.493	0.414	—	—	—
	**Subpleural Parenchymal Bands**			**Bronchiectasis**			**Honeycombing**			**Atelectasis**		
	**aOR**	**95% CI**	***p***	**aOR**	**95% CI**	***p***	**aOR**	**95% CI**	***p***	**aOR**	**95% CI**	***p***
Male	0.880	0.363–2.132	0.777	1.741	0.647–4.685	0.273	2.971	0.323–27.316	0.336	1.858	0.710–4.864	0.207
High flow oxygen therapy	12.612	1.483–107.287	0.020	—	—	—	0.000	0.000–NA	0.999	0.000	0.000–NA	0.999
Mechanical ventilation	—	—	—	3.022	0.514–17.761	0.221	1.666	0.000–NA	1.000	0.000	0.000–NA	0.999
Age ≥ 71 years	1.719	0.665–4.442	0.264	2.302	0.852–6.218	0.100	1.598	0.259–9.869	0.614	1.404	0.515–3.826	0.507
Peak ferritin ≥ 1728 µg/L	—	—	—	3.140	1.150–8.576	0.026	0.000	0.000–NA	0.998	—	—	—

Abbreviations: aOR, adjusted odds ratio; CI, confidence interval; CRP, C-reactive protein; NA, not available.

## Data Availability

All data generated or analyzed during this study are included in this published article.

## Abbreviations

The following abbreviations are used in this manuscript:

HRCT, high-resolution computed tomography; HFOT, high-flow oxygen therapy; WHO, World Health Organization; SARS-CoV-2, severe acute respiratory syndrome coronavirus 2; ACE-2, angiotensin-converting enzyme 2; DLCO, low diffusing capacity for carbon monoxide; RT-PCR, real-time polymerase chain reaction; ICU, intensive care unit; ARDS, acute respiratory distress syndrome; PaO_2_, partial pressure arterial oxygen; FiO_2_, fraction of inspired oxygen; CRP, C-reactive protein; IL, interleukin; SD, standard deviation; IQR, interquartile range; OR, odds ratio; CI, confidence interval; AEMPS, Spanish Agency for Medicines and Medical Devices; CPAP, continuous positive airway pressure; BMI, body mass index; mdn, median.
